# Supplementary material for: Curated and harmonised transcriptomics datasets of interstitial lung diseases
Source: Data Brief. 2025 Oct 14;63:112139. doi: 10.1016/j.dib.2025.112139 (PMC12581653; doi:10.1016/j.dib.2025.112139)
Supplement: Supplementary file 1 [file mmc1.zip › Supplementary_material/DNA-microarray/GSE10667/GSE10667_eUTOPIA_Agilent_QC_Report_2024-02-15.tar.gz!/Users/OWNER/AppData/Local/Temp/RtmpspbZX7/2024-02-15/ma.pdf]

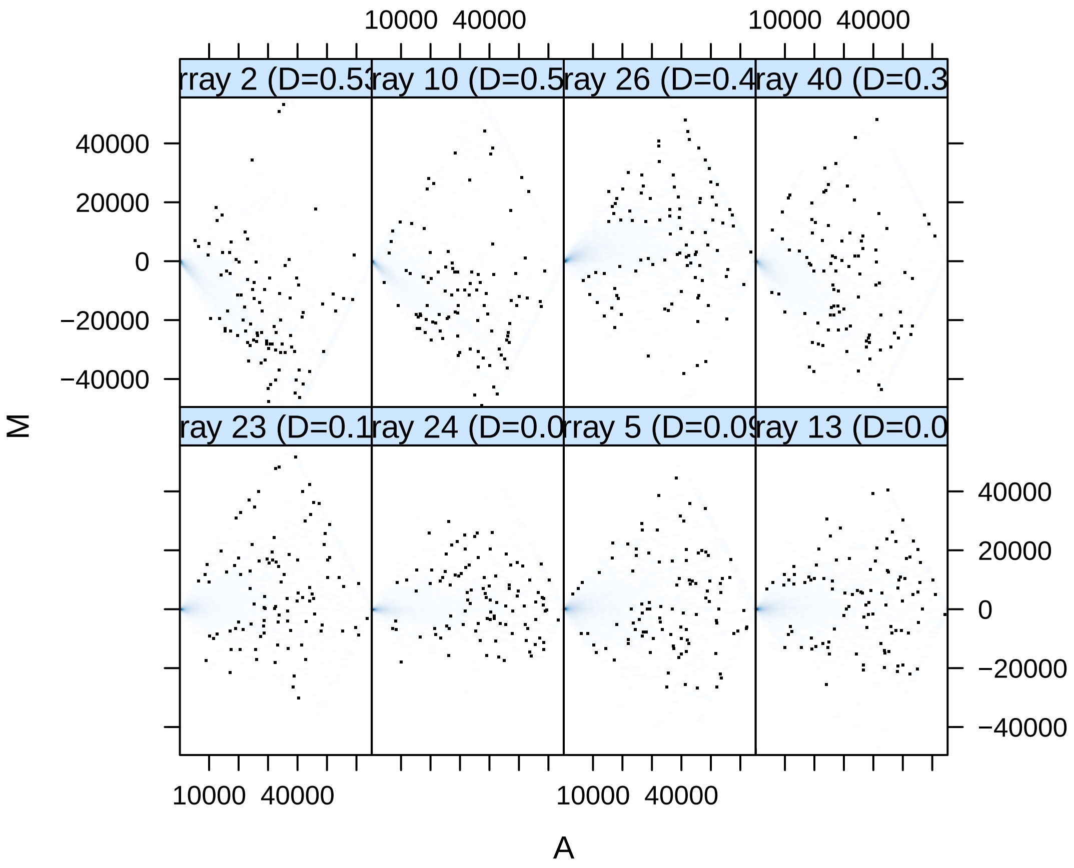

rray 2 (D=0.5
ray 10 (D=0.5
rray 26 (D=0.4
ray 40 (D=0.3
ray 23 (D=0.1
ray 24 (D=0.0
rray 5 (D=0.0
ray 13 (D=0.0
10000
40000
40000
20000
0
−20000
−40000
M
A
